# Non-myeloablative busulfan chimeric mouse models are less pro-inflammatory than head-shielded irradiation for studying immune cell interactions in brain tumours

**DOI:** 10.1186/s12974-019-1410-y

**Published:** 2019-02-05

**Authors:** A. Saam Youshani, Samuel Rowlston, Claire O’Leary, Gabriella Forte, Helen Parker, Aiyin Liao, Brian Telfer, Kaye Williams, Ian D. Kamaly-Asl, Brian W. Bigger

**Affiliations:** 1 Stem Cell and Neurotherapies Laboratory, Division of Cell Matrix Biology and Regenerative Medicine, School of Biological Sciences, Faculty of Biology, Medicine and Health, University of Manchester, Manchester, UK; 2 Department of Neurosurgery, Salford Royal Hospital, Salford, UK; 3 Division of Pharmacy and Optometry, School of Biological Sciences, Faculty of Biology, Medicine and Health, University of Manchester, Manchester, UK; 4 Department of Neurosurgery, Royal Manchester Children’s Hospital, Manchester, UK

**Keywords:** Chimeric mouse model, Head-shielded irradiation, Non-myeloablative conditioning, Inflammation, Glioblastoma, Macrophages, Microglia

## Abstract

**Background:**

Chimeric mouse models generated via adoptive bone marrow transfer are the foundation for immune cell tracking in neuroinflammation. Chimeras that exhibit low chimerism levels, blood-brain barrier disruption and pro-inflammatory effects prior to the progression of the pathological phenotype, make it difficult to distinguish the role of immune cells in neuroinflammatory conditions. Head-shielded irradiation overcomes many of the issues described and replaces the recipient bone marrow system with donor haematopoietic cells expressing a reporter gene or different pan-leukocyte antigen, whilst leaving the blood-brain barrier intact. However, our previous work with full body irradiation suggests that this may generate a pro-inflammatory peripheral environment which could impact on the brain’s immune microenvironment. Our aim was to compare non-myeloablative busulfan conditioning against head-shielded irradiation bone marrow chimeras prior to implantation of glioblastoma, a malignant brain tumour with a pro-inflammatory phenotype.

**Methods:**

Recipient wild-type/CD45.1 mice received non-myeloablative busulfan conditioning (25 mg/kg), full intensity head-shielded irradiation, full intensity busulfan conditioning (125 mg/kg) prior to transplant with whole bone marrow from CD45.2 donors and were compared against untransplanted controls. Half the mice from each group were orthotopically implanted with syngeneic GL-261 glioblastoma cells. We assessed peripheral blood, bone marrow and spleen chimerism, multi-organ pro-inflammatory cytokine profiles at 12 weeks and brain chimerism and immune cell infiltration by whole brain flow cytometry before and after implantation of glioblastoma at 12 and 14 weeks respectively.

**Results:**

Both non-myeloablative conditioning and head-shielded irradiation achieve equivalent blood and spleen chimerism of approximately 80%, although bone marrow engraftment is higher in the head-shielded irradiation group and highest in the fully conditioned group. Head-shielded irradiation stimulated pro-inflammatory cytokines in the blood and spleen but not in the brain, suggesting a systemic response to irradiation, whilst non-myeloablative conditioning showed no cytokine elevation. Non-myeloablative conditioning achieved higher donor chimerism in the brain after glioblastoma implantation than head-shielded irradiation with an altered immune cell profile.

**Conclusion:**

Our data suggest that non-myeloablative conditioning generates a more homeostatic peripheral inflammatory environment than head-shielded irradiation to allow a more consistent evaluation of immune cells in glioblastoma and can be used to investigate the roles of peripheral immune cells and bone marrow-derived subsets in other neurological diseases.

**Electronic supplementary material:**

The online version of this article (10.1186/s12974-019-1410-y) contains supplementary material, which is available to authorized users.

## Introduction

Adoptive transfer can be used to generate bone marrow chimeras and track immune cell populations in both homeostatic and inflammatory states. Optimal bone marrow (BM) transplants aim for high levels of donor chimerism, early engraftment, and minimal iatrogenic effects on animal health. An important aspect for the generation of a chimera is recipient conditioning prior to haematopoietic stem cell transplantation (HSCT). The pre-conditioning techniques commonly used are irradiation and chemotherapy [1]. Gamma ray irradiation induces host stem cell apoptosis, whilst busulfan, a DNA alkylating chemotherapy agent also induces cell death and consequent repopulation [2–5].

In the context of neurological diseases, the central nervous system (CNS) consists of a tight endothelial blood-brain barrier (BBB) that protects the brain from the systemic circulation [6]. Any treatment used to deplete recipient BM must maintain the integrity of the BBB to successfully achieve central or brain homeostasis in addition to minimising peripheral inflammation pre-BM transplantation. Notably, after HSCT, high levels of chimerism must also be achieved to accurately distinguish between recipient brain populations and donor peripheral immune cells and reliably determine respective contributions in a pathological state without concern about the negative effects of pre-conditioning.

Both irradiation and busulfan possess the unique property to deplete non-cycling primitive stem cells ensuring long-term donor BM chimerism [7]. Previous studies have compared whole-body irradiation (WBI) versus reduced intensity busulfan [8], WBI versus full conditioning (FC) busulfan [9], and WBI versus head-shielded irradiation (HIR) [10]. In all three studies, WBI established high peripheral blood chimerism levels, but adversely breached the BBB resulting in brain infiltration of peripheral immune cells and inadvertently stimulated a peripheral pro-inflammatory environment for up to 6 months post-transplant [8–10]. Furthermore, Schilling et al. achieved greater than 90% peripheral blood chimerism using a sub-lethal irradiation dose of 7 Gy and subsequently less BBB damage [11–14]. Head-shielded irradiation (HIR) was used to redress the issues of BBB damage and investigate the role of peripheral monocytic-derived macrophages (Mɸ) and resident brain tissue microglia in glioblastoma (GBM) and external autoimmune encephalomyelitis [15, 16]. In GBM, studies have proven HIR to be more effective and accurate in comparison to WBI, in an effort to quantify peripheral and central glioma-associated macrophages and microglia (GAMM) contributions [10, 17]. Nonetheless, what remains unclear is whether peripheral blood inflammation evident from WBI also occurs in HIR and whether this could impact downstream cytokine expression and immune cell infiltration into the brain.

Different doses of busulfan can be used to achieve either myeloablation with FC, reduced intensity or non-myeloablative conditioning (NMC) [9]. Previous studies have used busulfan at myeloablative doses intentionally aimed at stimulating transmigration of peripheral immune cells across the BBB and into the brain [18]. At lower doses of 90 μg/g busulfan can achieve high levels of chimerism and maintain an intact BBB whilst avoiding unwanted toxic side effects [8]. Thus, reduced intensity busulfan given at 30 μg/g body weight on days 7, 5 and 3 prior to BM transplant, demonstrated near equal chimerism of peripheral immune cell populations in comparison to WBI, minimal BBB breach and no frank myeloid cell engraftment to a non-diseased brain [8]. Busulfan provides an alternative technique to maximise chimerism and maintain homeostasis. To our knowledge, the NMC busulfan regimen (25 mg/kg), which is even lower than previously used examples has only been used in one study to investigate GAMM contribution in GBM [19].

What remains unknown is whether pre-conditioning with non-myeloablative busulfan or head-shielded irradiation inadvertently subjects the host to unwanted upregulation of circulating pro-inflammatory cytokines, resulting in reduced migrating BM cells into the brain post-BM transplant. Currently, we know that the BBB integrity is intact using the NMC model, evident by the absence of IgG and fibrinogen staining in the brain [19], whilst HIR mice also retain an unperturbed BBB post-transplant [10, 17]. However, no direct comparison exists between NMC and HIR models to determine the effects on brain immunophenotype as well as the pro-inflammatory cytokine expression in the brain and peripheral organs. Therefore, our aim was to investigate the effects of pre-conditioning on the host mouse physiology using three treatment groups, NMC busulfan, HIR and FC busulfan and an untransplanted control group (UnTx) prior to orthotopic intracranial implantation of GL-261 glioblastoma cell line.

## Methods

### Glioma cell culture

Mouse GBM cell line GL-261 was obtained from the National Institute of Cancer (Bethesda, USA), and cultured in Roswell Park Memorial Institute medium (RPMI-1640) (Sigma-Aldrich, Gillingham, UK) containing: 10% heat-inactivated foetal calf serum (FCS) and 2 mM L-glutamine with no antibiotics (GL-261 media).

### Animal maintenance, husbandry and care

All mice were housed in closed individually ventilated cages (IVC), in groups of 2 to 5 per cage and maintained at 21 ± 1 °C, with a constant humidity of 45–65%, on a 12-h light/dark cycle with ad libitum access to food and water. These studies were approved by the Ethics Committee of the University of Manchester and conducted under PPL40/3658 in accordance with the Animals Scientific Procedures Act, 1986 amendment regulations 2012.

### Animals

Female mice were used in all experiments due to housing restrictions. A previous study in our lab has shown no differences in inflammatory profiles between males and females when using busulfan [20]. C57BL/6J/CD45.2 mice (Envigo, Derby, UK) were termed wild-types (WT)/CD45.2, PEP-3/CD45.1 (B6.SJL-*Ptprc*^*a*^ *Pepc*^*b*^/BoyJ) mice bred in-house were congenic and comparative to WT and named WT/CD45.1.

### Bone marrow transplantation and peripheral blood chimerism

All transplant mice were age-matched from 8 to 10 weeks of age. Recipients were placed on a sterile diet of acidified water (pH 2.8) and gamma-irradiated food 7 days prior to busulfan dosing or irradiation treatment.

BM chimeric mice given non-myeloablative busulfan conditioning were generated as previously described [9, 19]. A single pre-conditioning dose of 25 mg/kg busulfan (Busilvex) (Pierre Fabre, Boulogne, France) was injected intraperitoneally 24 h prior to BM reconstitution. Fully conditioned mice were myeloablated to a maximum dose of 125 mg/kg busulfan divided into daily doses of 25 mg/kg injected intraperitoneally on five consecutive days prior to BM transplant. Whilst the head-shielded irradiation group were pre-conditioned with a total 11 Gy body irradiation (Ago HS X-ray System MP1, UK), split into two equal doses of 5.5 Gy given 3 h apart and the head protected using a lead shield.

Following pre-transplant conditioning all mice were injected with 3 × 10^7^ donor BM cells harvested from the pelvis, femur and tibia of syngeneic mouse, delivered via tail vein injection in 200 μL phosphate-buffered saline (PBS).

All mice that underwent a transplant were abbreviated as donor➔recipient. Throughout this study, WT/CD45.2➔WT/CD45.1 female mice transplants were performed.

Peripheral blood chimerism was analysed from tail vein sampling using flow cytometry BD FACS Canto II (BD). Blood samples were washed and prepared in 2% FCS/PBS buffer solution and underwent red blood cell lysis prior to staining with flow antibodies (Table 1). All samples were analysed using Flowjo v10 (FlowJo LLC., Ashland, OR, USA).

**Table 1:** Antibodies used for chimerism

Manufacturer	Fluorochrome conjugated	Antibody/antigen	Clone	Isotype	Dilution	Catalogue number
–	–	OKABE-eGFP (native GFP)	–	–	–	–
BD	PE	CD45.2	104	Mouse IgG2a, κ	1:400	560695
Thermo Fisher Scientific	APC	TOPRO-3	–	N/A	1:1000	T3605
Biolegend	APC-Cy7	CD45.1	A20	Rat IgG2a, κ	1:15	110715

### Stereotactic injection of GL-261 cells into intracranial compartment

Chimeric mice 12 weeks post-BM transplant and UnTx mice were anaesthetised and orthotopically intracranially implanted with 5 × 10^4^ GL-261 cells, as previously described [19]. The brains were harvested on day 14 post-surgery (*n* = 3/group). One mouse from the NMC group was excluded due to reflux of cells during intracranial injection.

### Brain sample dissociation

Dissociation of cerebral hemisphere was performed as previously described [19, 21]. The brain was divided into tumour-bearing and non-tumour-bearing hemispheres. Only the tumour-bearing hemisphere was used throughout this study, because contralateral hemispheres have shown inflammatory profiles and cannot be used as controls [19].

Whole brain dissociation into single cell suspension was adapted from Robinson et al. with key modifications [21]. Different concentration of Percoll® (P1644) (Sigma-Aldrich, Dorset, UK) were set up by first mixing nine parts Percoll with one part of 10× PBS (Thermo Fisher Scientific, Loughborough, UK). Final concentrations of 35% and 70% Percoll were then constituted with standard 1× PBS.

Terminal perfusion via the left ventricle was performed using cold PBS/5 mM EDTA (Sigma-Aldrich, Dorse, UK). The tumour-bearing hemisphere was then divided from the non-tumour-bearing hemisphere and processed in 1 mL Accutase (Merck Millipore, Darmstadt, Germany) for 25 min at 37 °C. Enzymatic reaction was diluted with 5 mL of 2% FCS/2 mM EDTA/PBS (FEP) and samples mechanically dissociated through a 100 μm cell strainer (Corning, New York, NY, USA). The cell strainer was washed with FEP until clean and centrifuged at 280×*g* for 7 min at 6 °C. The supernatant was discarded and resuspended in 6 mL 35% Percoll and underlaid with 2 mL 70% Percoll. The sample was centrifuged at 650×*g* without brake for 15 min at room temperature. The myelin layer was carefully aspirated and a thin ‘milky layer’ of cells at the 35%/70% interface was aspirated and washed with 5 mL of FEP. The cell suspension was centrifuged at 300×*g* for 5 min at 6 °C and cell pellet resuspended in 200 μL 2% FCS/PBS in preparation for flow cytometry.

### Cell preparation and analysis using flow cytometry

Cells were counted, stained and prepared for flow cytometry as previously described [19]. Antibodies used for staining are shown in Table 2, FlowJo v10 was used to analyse all samples.

**Table 2:** Antibodies used to immunophenotype brain samples

Manufacturer	Fluorochrome conjugated	Antibody/antigen	Clone	Isotype	Dilution	Catalogue number
Biolegend	AF488	PDCA-1	927	Rat IgG2b, κ	1:25	127012
Biolegend	PerCP/Cy5.5	CX3CR1	SA011F11	Mouse IgG2a, κ	1:25	149009
Miltenyi Biotec	APC	MerTK	REA477	Recombinant human IgG1	1:5	130-107-479
Biolegend	AF700	Ly6G	1A8	Rat IgG2a, κ	1:15	127621
eBiosciences	APC/eFluor780	CD3	17A2	Rat IgG2b, κ	1:50	47-0032-82
eBiosciences	APC/eFluor780	CD19	eBIO1D3	Rat IgG2b, κ	1:75	47-0193-82
BD	BV421	Siglec-F	E50-2440	Rat IgG2a, κ	1:150	562681
Biolegend	BV510	Ly6C	HK1.4	Rat IgG2c, κ	1:300	128033
Biolegend	BV650	CD45.1	A20	Mouse (A.SW) IgG2a, κ	1:75	110735
Biolegend	BV711	CD11b	M1/70	Rat IgG2b, κ	1:100	101241
Life Technologies	Live/dead blue	Cellular protein (amines)	–	–	1:400	L23105
Biolegend	PE	Siglec-H	551	Rat IgG1, κ	1:25	129606
Biolegend	PE/CF594	CD45.2	104	Mouse (SJL) IgG2a, κ	1:200	109845
Biolegend	PE/Cy5	MHCII	M5/114.14.2	Rat IgG2b, κ	1:6000	107611
Biolegend	PE/Cy7	CD64	X54-5/7.1	Mouse IgG1, κ	1:40	139313

### Antibody panel used for brain flow

#### Quantitative PCR and gene expression analysis

RNA was extracted from the brain and spleen at 12 weeks post-BM transplant. RNA was extracted from the blood at 2 and 12 weeks post-BM transplant. RNA isolation was performed using Trizol® Reagent (Trizol) (Life Technologies). All tissue samples were homogenised in 1 mL of Trizol (per 50-100 mg of tissue) until complete dissociation of nucleoprotein complexes and incubated at room temperature for 5 min. For every 1 mL of Trizol, 200 μL chloroform was added and vortexed on full speed for approximately 10 s to form a homogenous ‘pale pink’ mixture. The samples were incubated for 2 min at room temperature and centrifuged at 12000×*g* for 15 min at 4 °C. Following centrifugation, a 3-layered density gradient was seen; the upper aqueous phase containing RNA was aspirated and transferred to a sterile 1.5 mL tube. Approximately 0.5 mL of isopropanol was added per 1 mL of Trizol reagent and mixed thoroughly in order to precipitate the RNA. Samples were incubated for 10 min at room temperature and centrifuged at 12000×*g* for 10 min at 4 °C. The RNA precipitate formed a pellet on the bottom of the tube. The supernatant was removed, and RNA pellet was washed once with 1 mL of ice-cold 75% ethanol. The mixture was vortexed gently and centrifuged at 7500×*g* for 5 min at 4 °C. Typically, the RNA pellet became clear and the supernatant was removed carefully to remove all traces of ethanol and the pellet allowed to air-dry. The final cell pellet was suspended in 20 μL molecular-grade H_2_O ‘Hyclone’ (GE Healthcare Life Sciences, Hatfield, UK) and stored at − 80 °C. Samples were treated with DNase using the “Turbo DNA-free” kit (Life Technologies) and 1 μg of RNA was transcribed into cDNA using the “High-Capacity cDNA Reverse Transcription Kit” (Applied Biosystems, Warrington, UK).

#### Taqman™ gene expression assays kit

As per manufacturer guidelines, Taqman™ gene expression assays (Applied Biosystems, Warrington, UK) containing sequence-specific mouse primers for tumour necrosis factor alpha (TNF-α) (Assay ID: Mm00443258_m1 *Tnfa*), interleukin-1 beta (IL-1β) (Assay ID: Mm00434228_m1 *Il1b*), monocyte chemoattractant protein-1 (MCP-1/CCL2) (Assay ID: Mm00441242_m1 *Ccl2*), interleukin-6 (IL-6) (Assay ID: Mm00446190_m1 *Il6*) and interferon gamma (IFN-ɣ) (Assay ID: Mm01168134_m1 *Ifng*) Interleukin-10 (IL-10) (Assay ID: Mm00439616_m1 *Il10*), interferon alpha 4 (IFN-α4) (Assay ID: Mm00833969_s1 *Ifna4*), chemokine (C-X-C motif) ligand 10 (CXCL10) (Assay ID: Mm00445235_m1 *Cxcl10*), interleukin-4 (IL-4) (Assay ID: Mm00445259_m1 *Il4*) were used to achieve target specific amplification. Glyceraldehyde 3-phosphate dehydrogenase (GAPDH) (Assay ID: 99999915_g1 *Gapdh*) was used as the endogenous housekeeping gene.

Quantitative real-time polymerase chain reaction was performed using StepOnePlus Real-Time PCR System (Applied Biosystems). The cycling parameters used were1 cycle of 50 °C for 2 min followed by 95 °C for 10 min, then 40 cycles of 95 °C for 15 s and 60 °C for 60 s. Samples were stored at 4 °C if used the same day and − 20 °C if required at a later date.

All samples were analysed in duplicates. Non-template controls (molecular-grade H_2_O) were included to assess contamination. Naïve non-treated mouse tissues were used as reference controls. For each gene of interest, the mean ΔCT of the control samples was used to subtract from ΔCT of experimental samples (ΔΔCT). RQ values were calculated as 2^(−ΔΔCT) and RQ normalised by dividing the RQ value for each gene by the average value of all the control samples.

### Statistical analysis

Statistical analysis was performed using the software package GraphPad Prism 7 (version 7.03) (GraphPad Software, USA). Statistical significance level was defined as an alpha of 0.05. Shapiro-Wilk normality test was used to analyse raw gene expression values. Samples found failing the normality test were analysed using the non-parametric Kruskal-Wallis test with Dunn’s post hoc correction for multiple comparisons. Otherwise, samples were analysed using one-way ANOVA with Tukey’s post hoc correction for multiple comparisons. For gene expression analysis, if more than a fivefold difference was detected between samples and variance was unequal, data was log transformed to equalise variances and improve analysis. Two-way ANOVA was used for all other samples comparing two or more variable in two or more groups with Tukey’s post hoc correction for multiple comparisons.

For flow cytometry data, results were reported as the mean percentage proportion of the experiments per antibody panel.

Mice with CD45.1/CD45.2 chimerism < 70% at 12 weeks produced too much variability for downstream analysis of central and peripheral immune cell populations in GBM and thus were excluded (typically < 5%).

## Results

### Non-myeloablative conditioning achieves equivalent blood chimerism to head-shielded irradiation

**Fig. 1:**
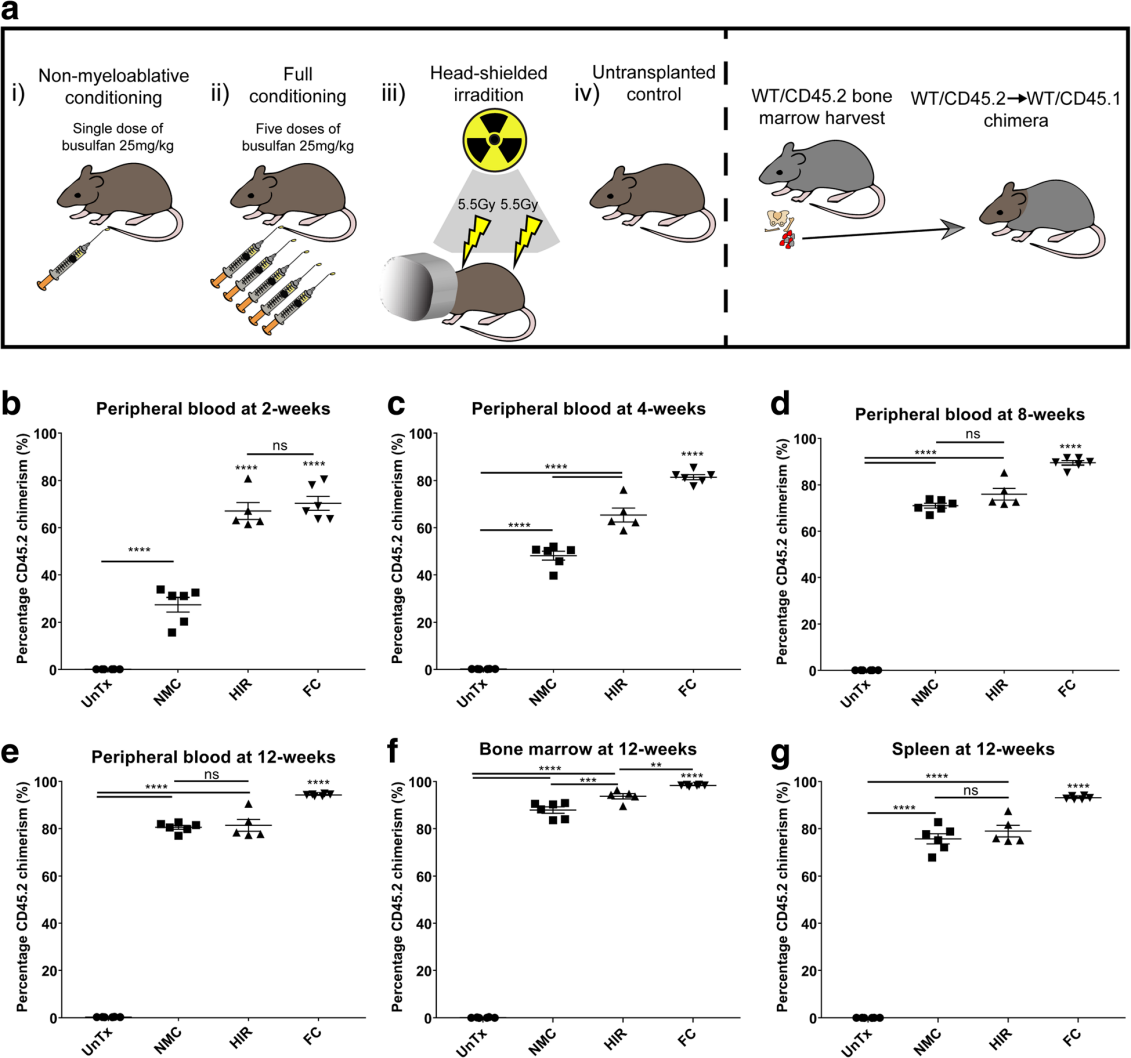
Peripheral chimerism analysis of transplanted mice after different pre-conditioning regimens. **a** Four groups received different pre-conditioning regimens: (i) non-myeloablative conditioning (NMC) using a single dose of busulfan at 25 mg/kg (*n* = 6/group), (ii) full conditioning (FC) busulfan 125 mg/kg given over five consecutive days (*n* = 6/group), (iii) head-shielded irradiation (HIR) at a divided dose of 11 Gy given 3 h apart (*n* = 6/group) and (iv) untransplanted controls (UnTx) (*n* = 6/group). **b** Peripheral blood chimerism at 2 weeks post-transplant, **c** 4 weeks post-transplant, **d** 8 weeks post-transplant and **e** 12 weeks post-transplant. **f** Bone marrow chimerism measured at 12 weeks post-transplant and **g** spleen chimerism at 12 weeks. One-way ANOVA with Tukey’s post hoc correction for multiple comparisons was used. Error bars represent the SEM. ****p < 0.0001; ***p < 0.001; **p < 0.01; *p < 0.05; ns = non-significant

Having shown previously [19] that the NMC model can generate high levels of chimerism without damaging the BBB, we compared our chimeric model with the commonly used HIR myeloablative technique to determine if either model was superior in terms of peripheral blood chimerism of donor haematopoietic cells. An UnTx control group and FC busulfan group were used as comparative controls throughout the study (Fig. 1). One mouse was excluded from the head-shielded group, after inadvertently receiving a head irradiation dose.

We transplanted WT/CD45.2 BM into WT/CD45.1 mice (*n* = 6/group) and successively measured peripheral blood CD45.2 donor chimerism using flow cytometry at 2, 4, 8 and 12 weeks. At 2 weeks post-BM transplant, HIR (67.1%) and FC (70.4%) showed superior levels of chimerism (*p* < 0.0001) compared to NMC chimerism of 27.4% and controls with 0.1% (Fig. 1b).

At 4 weeks post-transplant, both busulfan pre-conditioned groups resulted in increased donor chimerism with NMC at 48.2% and FC at 81.5% (Fig. 1c). The HIR group appeared to plateau at 65.4% donor CD45.2 chimerism (Fig. 1c). Nonetheless, a significant difference was noted when NMC was compared to both HIR and FC groups (*p* < 0.0001) (Fig. 1c). FC was also now significantly different to HIR (*p* < 0.0001) (Fig. 1c).

At 8 weeks after BM reconstitution, HIR showed 76% engraftment and was no longer significantly different to NMC chimerism 71.1%. FC showed the highest donor chimerism levels at 89.5% (Fig. 1d).

At the final chimerism check 12 weeks post-transplant, there was no significant difference in donor engraftment between NMC of 80.6% and HIR of 81.4%; FC however significantly differed to both groups with an overall chimerism of 94.3% (Fig. 1e). In both HIR and FC busulfan mice, there was obvious macroscopic damage to skin melanocytes with a fur phenotypic appearance changing from black to grey.

Comparing all four groups over time shows a trend of increasing NMC donor chimerism that equals HIR chimerism by 12 weeks (Additional file 1: Figure S1A-B).

We also investigated chimerism in haematogenic organs integral to successful engraftment, these included the BM and spleen (Fig. 1f, g). All samples were processed at 12 weeks post-transplant (Additional file 2: Figure S2).

In the BM, as a percentage of total CD45-positive cells, HIR and FC groups showed the highest engraftment. HIR showed 93.8% CD45.2^+^ donor cell chimerism and FC showed 98.4%, both significantly differed to the NMC group which had an average donor engraftment of 87.9% (Fig. 1f). In the spleen, no differences were noted in donor chimerism between NMC (75.7%) and HIR (79%). FC showed the overall highest donor engraftment of 93.2% with a significant difference with all other groups (*p* < 0.0001) (Fig. 1g).

### Head-shielded irradiation is pro-inflammatory in peripheral blood 12 weeks post-transplant

After establishing chimerism in all four groups, we wanted to determine the baseline homeostatic state in all four groups after BM transplant, but prior to GBM implantation (*n* = 6/group). We tested peripheral blood at 2 and 12 weeks post-BM transplant for four pro-inflammatory cytokines: *Il1b*, *Ccl2*, *Il6* and *Tnfa*.

**Fig. 2:**
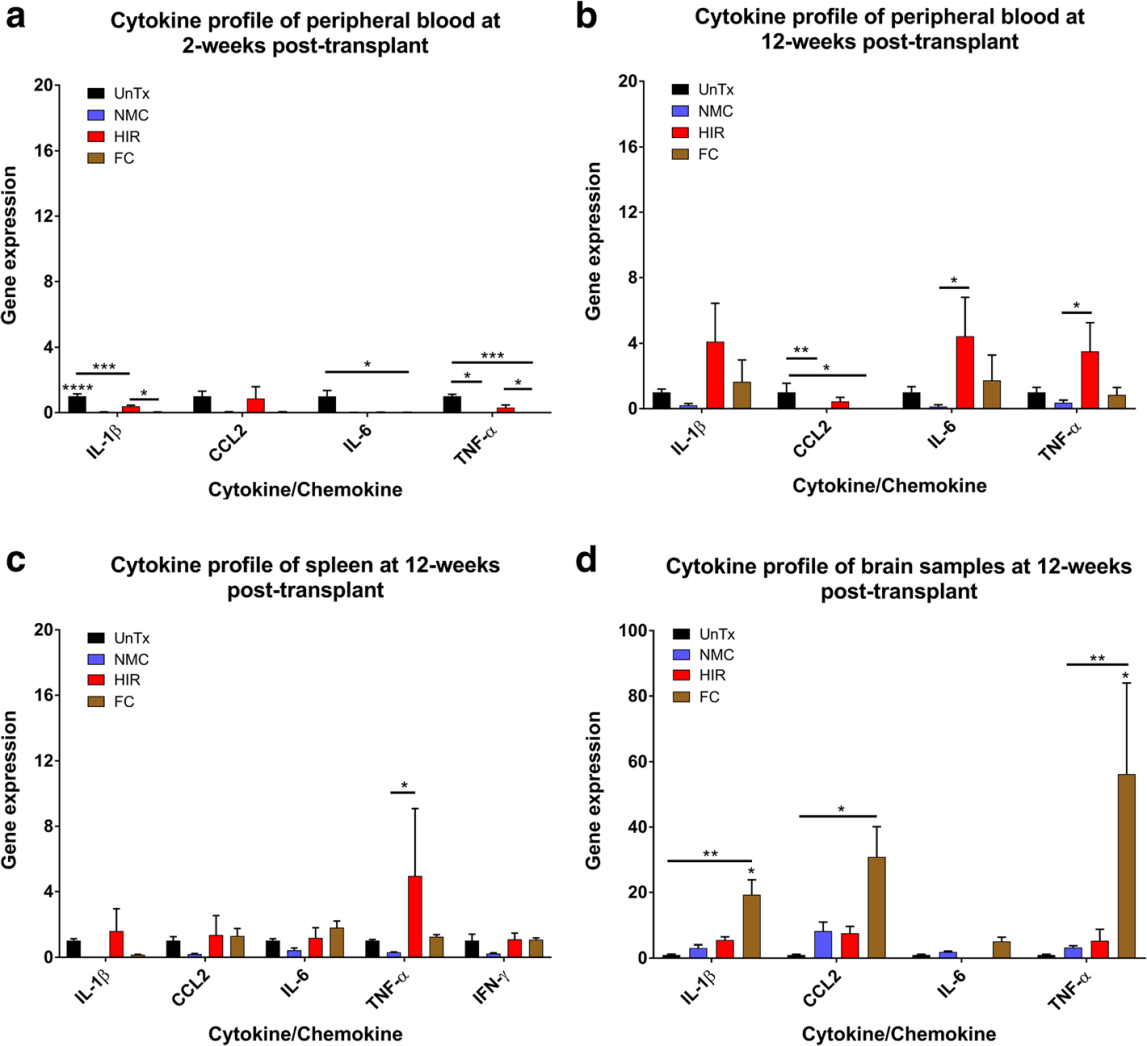
Gene expression of peripheral blood at 2 weeks and peripheral blood, spleen and brain at 12 weeks. **a** Gene expression of peripheral blood samples taken at 2 weeks post-transplant and tested for *Il1b*, *Ccl2*, *Il6* and *Tnfa* (*n* = 6/group). **b** Gene expression of peripheral blood samples taken at 12 weeks post-transplant and tested for *Il1b*, *Ccl2*, *Il6*, and *Tnfa* (*n* = 6/group). **c** Spleen sample gene expression profiles were measured for the cytokines *Il1b*, *Ccl2*, *Il6*, *Tnfa* and *Ifng*. **d** Brain samples without a tumour were analysed for the cytokines *Il1b*, *Ccl2*, *Il6* and *Tnfa*. *Ccl2*, *Tnfa* and *Il6* expression in blood at 2 weeks, *Il1b*, *Ccl2*, *Tnfa* and *Il6* expression in the blood at 12 weeks, *Il6* expression in the spleen and *Ccl2* expression in the brain failed Shapiro-Wilk normality test and were analysed using Kruskal-Wallis test with Dunn’s post hoc correction for multiple comparisons. All other samples were analysed using one-way ANOVA with Tukey’s post hoc correction for multiple comparisons. Error bar represents the SEM. ****p < 0.0001; ***p < 0.001; **p < 0.01; *p < 0.05. Samples with ≥ 5-fold difference in gene expression and unequal variance were log transformed (spleen; *Tnfa*, brain; *Il1b*, *Ccl2* and *Tnfa*). Error bar represents the SEM. ****p < 0.0001; ***p < 0.001; **p < 0.01; *p < 0.05

At 2 weeks, as expected, we saw initial downregulation of all four pro-inflammatory genes in busulfan-conditioned and irradiated mice (Fig. 2a). *Il1b* was significantly downregulated in NMC and FC groups (*p* < 0.0001) and HIR chimeric mice (*p* < 0.001) compared to controls. HIR mice also had a higher expression of *Il1b* relative to FC mice (*p* < 0.05). *Tnfa* was significantly downregulated in both FC mice (*p* < 0.001) and less so in NMC mice (*p* < 0.05) against controls (Fig. 2a). *Il6* was also significantly downregulated in FC mice (*p* < 0.05) in comparison to controls (Fig. 2a).

At 12 weeks, post-BM transplant, an overall trend of increased pro-inflammatory gene expression *Il1b*, *Tnfa* and *Il6* was observed in the HIR transplant group (Fig. 2b). However, only *Il6* and *Tnfa* were significantly upregulated in the HIR group in comparison to NMC mice (*p* < 0.05) (Fig. 2b). Compared to controls, *Ccl2* expression was significantly downregulated in NMC (*p* < 0.01) and FC mice (*p* < 0.05) (Fig. 2b). No other significant differences were noted between all four transplant groups (Fig. 2b).

To determine other sources of pro-inflammation that could impact on immune cell infiltration, we tested the spleen and brain. Three spleen and brain samples were processed as the remaining three mice were implanted with GBM for flow cytometry analysis (Fig. 2).

Spleen samples in the HIR group showed a significant upregulation of *Tnfa* expression (*p* < 0.05) relative to NMC and an increasing trend in comparison to both control and FC groups (Fig. 1c). In the spleen, no other significant changes in gene expression were seen relative to control (Fig. 1c). NMC demonstrated an overall downregulation of all five genes relative to control, but no significant differences were noted (Fig. 1c).

Brain samples showed a significant increase in expression of *Il1b* in the FC group when compared with controls (*p* < 0.01) and both NMC (*p <* 0.05) and HIR (*p* < 0.05) groups (Fig. 1d). Furthermore, there was significant upregulation of the cytokine *Tnfa* in the FC group in comparison to controls (*p* < 0.01) and both NMC (*p* < 0.05) and HIR (*p* < 0.05) groups (Fig. 1d). *Ccl2* expression was significantly higher in the FC group compared against controls (*p* < 0.05) (Fig. 1d). No significant differences or trends in up- or downregulation of genes were seen between controls, NMC and HIR groups (Fig. 1d). Brain samples were also analysed for the gene expression of anti-inflammatory cytokines: *Il4*, *Il10*, *Cxcl10* and *Ifna4*. Overall, no significant changes were noted, although there was a higher expression of *Cxcl10* in both HIR and FC mice groups compared to controls and NMC; in addition, downregulation HIR mice showed downregulation of *Il10*. Untransplanted and NMC mice showed the greatest similarity. However, in all groups including controls, no expression of *Il4* was noted (Additional file 3).

### Brain immune cell proportions remain similar in non-myeloablative and head-shielded irradiation groups with and without brain tumours

At 12 weeks, all mouse groups were either injected with GL-261 GBM cells to generate brain tumours (*n* = 3) and analysed at 14 weeks; un-injected mice were used as controls at 12 weeks (*n* = 3) (Fig. 2a). Many groups use the non-tumour-injected brain hemisphere as a control for tumour, but we have previously shown that the BBB is breached after tumour implantation and the un-injected hemisphere is significantly compromised with immune cell infiltration. Mock injections give similar infiltration profiles to un-injected controls; therefore, we used the latter [19].

**Fig. 3:**
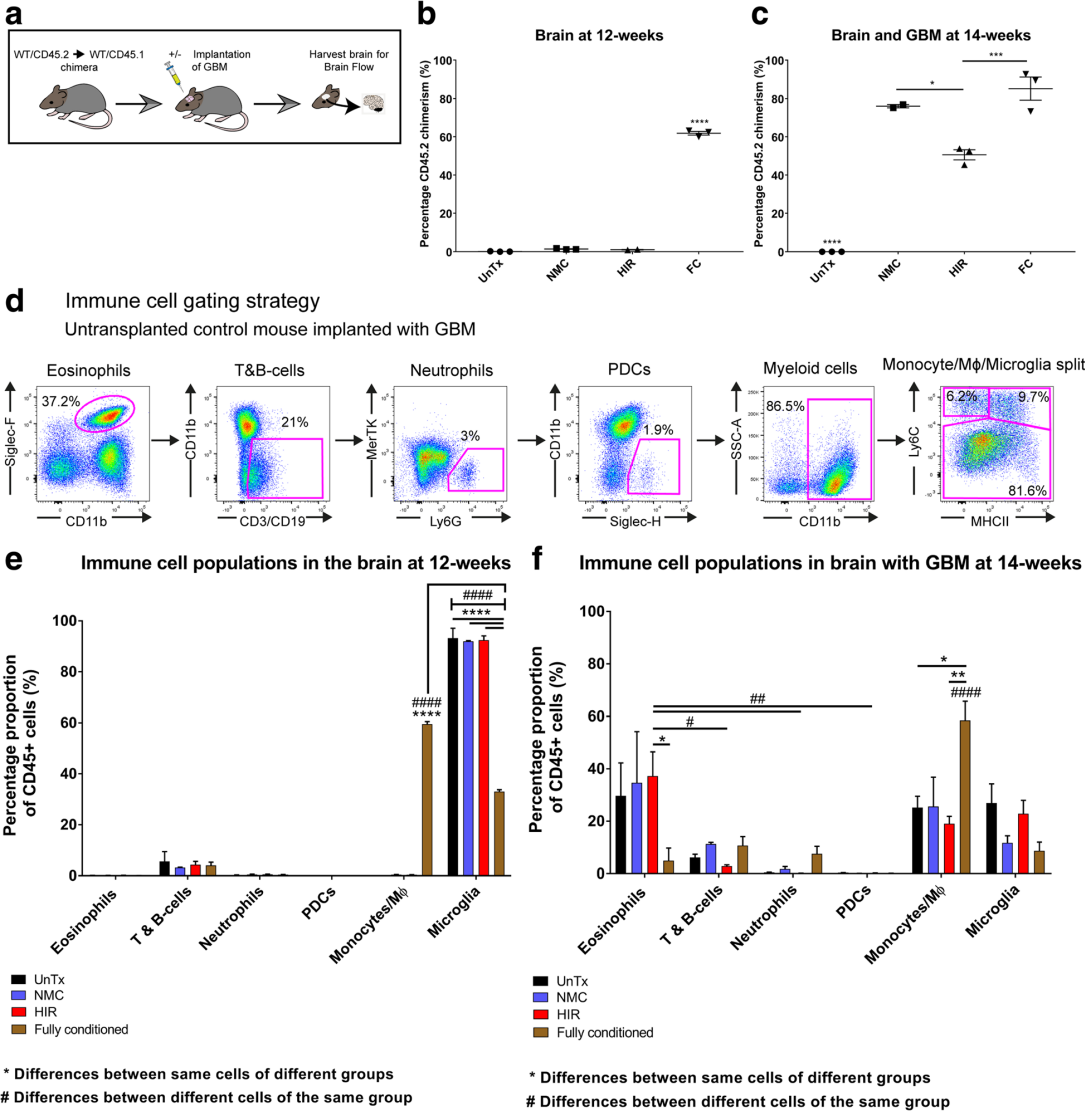
Brain immunophenotyping of different transplant groups with and without GBM**.** Brain immune cells were identified and quantified using flow cytometry. **a** Schematic diagram demonstrating how mouse brain samples in different groups were processed by brain digestion and flow cytometry with (*n* = 3/group) or without (*n* = 3/group) a brain tumour. **b** Naïve brain chimerism at 12 weeks, measured by calculating total CD45.2 cell engraftment. **c** Brain chimerism of mice implanted with GBM at 14 weeks, measured by calculating total CD45.2 engraftment. One-way ANOVA analysis with Tukey’s post hoc correction for multiple comparisons was used for all groups. Error bars represent the SEM. ****p < 0.0001; ***p < 0.001; *p < 0.05. **d** Gating strategy used to identify immune cell populations within the brain. **e** Immune cell profile, within the brain of different chimeric mice 12 weeks post-transplant (*n* = 3/group) without GBM. **f** Tumour-tropic immune cells found within the tumour-bearing hemisphere of different chimeric mice implanted with GBM (*n* = 3/group). Two-way ANOVA analysis with Tukey’s post hoc correction for multiple comparisons was used throughout. Error bar represents the SEM. **** or ^####^*p* < 0.0001; *** or ^###^*p* < 0.001; ** or ^##^*p* < 0.01; * or ^#^*p* < 0.05; ns = non-significant. *Differences between the same type of immune cell amongst different transplant groups. ^#^Differences between different immune cells within the same transplant group. One mouse from the NMC group was excluded due to reflux of tumour cells during surgery, thus providing unreliable downstream analysis

Naïve brains with no implanted tumour showed on average 1.4% donor cell infiltration in the NMC group and 1% donor CD45.2 engraftment in the HIR transplant, suggesting minimal damage to the BBB in either case (Fig. 2b and Additional file 2: Figure S2). UnTx controls, as expected, did not stain positive for CD45.2 (Fig. 3b and Additional file 2: Figure S2). The FC group showed 61.9% donor brain engraftment and significantly differed to all other groups (*p* < 0.0001) (Fig. 3b and Additional file 2: Figure S2). After GBM implantation, brain chimerism showed significantly increased donor cell chimerism in all transplant groups, with 76.1% donor cell engraftment in NMC, 50.6% in HIR and 85.2% in FC (Fig. 3c and Additional file 2: Figure S2). One mouse from the NMC group was excluded due to reflux of GL-261 cells at time of implantation.

We also quantified relative contributions of different immune cell subtypes using a method we previously developed to enable distinction between resident and donor myeloid populations [19] including eosinophils, B and T cells, neutrophils, plasmacytoid DCs, monocyte/Mɸ and resident microglia. The gating strategy outlined in Fig. 3d was used uniformly for all groups and throughout this study.

In the naïve brains prior to GBM implantation of UnTx controls, NMC and HIR groups, no differences were seen throughout all cell populations and an overall similar pattern of immune cell infiltration was noted (Fig. 3e). In all three groups, T and B cells represented the only peripherally infiltrating population with an average contribution of 5.6% in controls, 3.2% with NMC and 2.9% with HIR as a proportion of total CD45^+^ cells (Fig. 3e). In the FC group, T and B cells contributed to a total of 4.1%; however, there was a significant increase in monocytes/Mɸ (*p* < 0.0001) making up an average of 59.5% of the total CD45^+^ population. There was negligible monocyte-Mɸ infiltration in controls, NMC and HIR groups. Microglia represented the highest overall CD45^+^ percentage proportion (*p* < 0.0001). Furthermore, all three treatment groups showed an overall significant difference in microglia contribution in comparison to the FC transplanted group (*p* < 0.0001) (Fig. 3e).

The GBM-implanted brain displayed a different overall pattern of immune cell infiltration. The FC group showed a significant contribution of monocytes/Mɸ (58.5%) relative to HIR (19.1%; *p* < 0.05) and UnTx controls (25.2%; *p* < 0.01); no significant difference was noted with NMC (25.6%) (Fig. 3f). A comparison of immune cells within the FC group showed differentiated Mɸ as the most dominant population (*p* < 0.0001) (Fig. 3f). In HIR, NMC and controls, eosinophils were the most dominant population with an average contribution of 37.3%, 34.7% and 29.7% respectively (Fig. 3f). Eosinophils in the HIR group showed a significant difference compared with FC mice (5%; *p* < 0.05). In both controls and NMC, no statistical differences were noted between all cell types (Fig. 3f). The contribution of microglia in GBM-implanted mice was reduced in all four groups in comparison to mice without tumours, demonstrating a peripheral immune response in GBM.

Overall, the immunophenotypes of the untransplanted control and NMC groups were similar, which in comparison to the FC group showed a high eosinophilic response as a proportion of CD45-positive cells and a larger contribution of differentiated Mɸ as a proportion of monocytes/Mɸ.

### Head-shielded irradiation shows reduced peripheral myeloid contribution in the brain of GBM-implanted mice

**Fig. 4:**
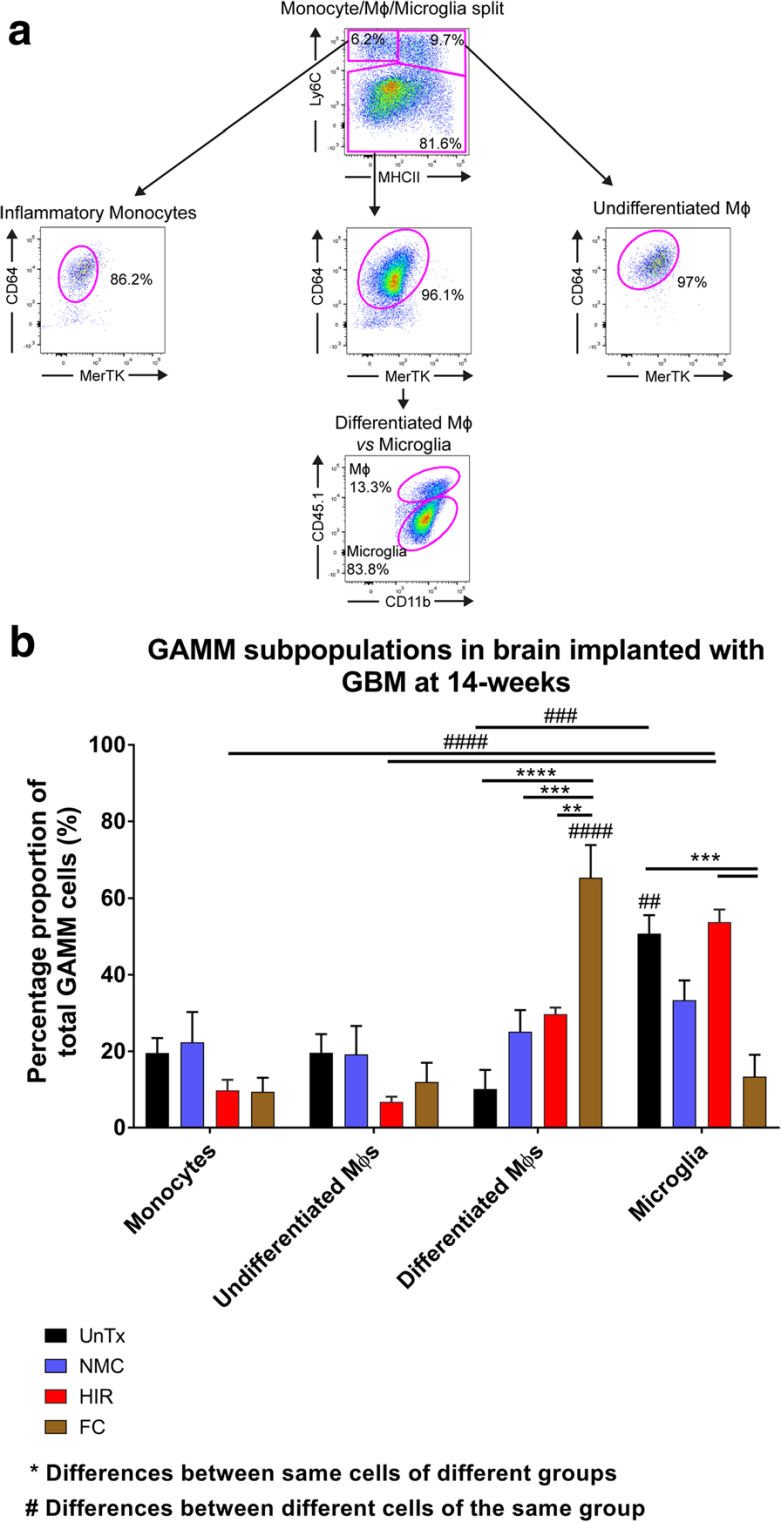
Monocytes, macrophages and microglia subpopulation analysis in the brain prior to and after GBM implantation**. a** Following the cytometry gating used in Fig. 2d, the remaining myeloid subpopulations of monocytes, macrophages (Mɸ) and microglia were further separated using Ly6C/MHCII/MerTK/CD64 into monocytes, undifferentiated Mɸ and a combination of differentiated Mɸ and microglia. The combined group of differentiated Mɸ and microglia were separated using CD11b/CD45. All brain samples were analysed using flow cytometry (*n* = 3/group). **b** Bar graph showing the relative glioma-associated macrophages and microglia (GAMM) subpopulations in tumour-bearing hemisphere of GBM-implanted mice. All samples were analysed using a two-way ANOVA with Tukey’s post hoc correction for multiple comparisons. Error bar represents the SEM. **** or ^####^*p* < 0.0001; ***or ^###^*p* < 0.001; **or ^##^*p* < 0.01. One mouse from the NMC group was excluded due to reflux of tumour cells during surgery, thus providing unreliable downstream analysis

GAMM myeloid subpopulations were calculated as a proportion of total GAMM cells, gating for monocytes, undifferentiated Mɸ, differentiated Mɸ and resident microglia as previously described [19] (Fig. 4a). Essentially, the four marker set Ly6C/MHCII/MerTK/CD64 distinguished between monocytes, undifferentiated Mɸ and a combined group of differentiated Mɸ and microglia (Fig. 4a). The group of differentiated Mɸ and microglia was later separated using the markers CD11b/CD45 (Fig. 4a).

Taking this gating strategy forward, we initially compared NMC and HIR transplant groups and showed no significant differences. Interestingly, a trend was noted, which illustrated a higher proportional representation of resident microglia in HIR mice (53.7%) compared to NMC mice (33.3%) (Fig. 4b). Analysis of the peripheral myeloid GAMM subpopulations in NMC transplants conversely showed an even distribution of peripherally infiltrating GAMM cells: monocytes 22.4%, undifferentiated Mɸ 19.2% and differentiated Mɸ 25.1%, whilst HIR mice consisted of monocytes 9.8%, undifferentiated Mɸ 6.8% and differentiated Mɸ 29.7% (Fig. 4b).

The aim was to achieve similar percentage proportions found in UnTx mice: microglia 50.8%, monocytes 19.6%, undifferentiated Mɸ 19.6% and differentiated Mɸ 10.1% (Fig. 4b). Thus, when comparing NMC with UnTx mice and HIR with UnTx mice, no significant differences were noted between either comparisons. Furthermore, in addition to the percentage proportions of total GAMM cells, calculating individual peripheral GAMM subpopulation (monocytes, undifferentiated Mɸ and differentiated Mɸ) contribution as a proportion of total monocytes, undifferentiated Mɸ and differentiated Mɸ showed an even distribution between all three GAMM subpopulations in NMC mice compared with UnTx mice: NMC monocytes 33.6%, undifferentiated Mɸ 28.8% and differentiated Mɸ 37.6% compared with UnTx control monocytes 39.8%%, undifferentiated Mɸs 39.8% and differentiated Mɸ 20.5% (Fig. 4b). However, in HIR transplants relative monocyte/Mɸ contribution was 21.2% monocytes, 14.7% undifferentiated Mɸ and 64.1% differentiated Mɸ, showing a bias towards matured Mɸ. In the FC transplant group, monocytes contributed 10.8%, undifferentiated Mɸ 13.8% and differentiated Mɸ 75.3% (Fig. 4b). Total number of cells did not differ between all four groups, highlighting the NMC subtype profile to be in keeping with UnTx mice compared to HIR.

Of note, was an overall higher propensity to attract differentiated Mɸ in the FC (65.3%) group. A significant difference was noted between FC differentiated Mɸ in comparison with all other transplant groups: HIR (29.7%; *p* < 0.01), NMC (25.1%; *p* < 0.001) and UnTx (10.1%; *p* < 0.0001) (Fig. 4b). FC microglia contributed significantly less (*p* < 0.001; 13.4%) compared with UnTx (50.8%) and HIR (53.8%) groups, with no significant differences noted with the NMC mice compared to all other groups (33.3%) (Fig. 4b).

## Discussion

We compared non-myeloablative conditioning (NMC) against head-shielded irradiation (HIR) chimeric transplant models using controls of full conditioning (FC) and a untransplanted group to determine peripheral chimerism, brain immune cell infiltration and pro-inflammatory gene expression. We subsequently implanted GBM to determine if the brain immune engraftment profile was changed between the four groups in a pro-inflammatory environment. To date, no study has compared the effects of NMC busulfan against HIR.

In neurological disease research, WBI [9, 22, 23] has been replaced by HIR as the gold standard, overcoming many of the WBI side-effects that include a sustained pro-inflammatory environment expressing IL-1α and perturbation of the BBB [10, 16]. We have previously used the NMC busulfan model to distinguish between central and peripheral myeloid cells and reliably achieved high levels of chimerism without damaging the BBB, as shown by using IgG and fibrinogen staining [19]. Comparing peripheral blood chimerism of both models showed effective donor engraftment after bone marrow transplantation in both HIR and NMC groups, achieving ≥ 80% peripheral blood chimerism and avoiding peripheral infiltration of immune cells within the brain. Comparatively, FC busulfan mice reached 94% peripheral blood chimerism, but subsequently disrupted the BBB promoting Mɸ infiltration [9]. This could be explained by the upregulation of pro-inflammatory cytokine MCP-1 in the serum and brain at 6 months post-treatment [9]. Although, we did not use WBI mice in this study, peripheral blood chimerism reaches similar levels to FC mice within 2 weeks, similar to HIR. Peripheral blood chimerism in NMC busulfan mice typically takes longer to reach 80% chimerism reaching a final chimerism plateau at 12 weeks. Both NMC and HIR mice demonstrated > 70% spleen chimerism 12 weeks post-BM transplant, but HIR produced a significantly higher overall BM engraftment compared to NMC (≥ 90% versus 85% BM chimerism).

At 2 weeks, there was downregulation of all pro-inflammatory cytokines (*Il1b*, *Ccl2*, *Il6* and *Tnfa*) within all adoptive transfer groups in comparison to UnTx controls. Interestingly, there was a greater significant downregulation in both busulfan-conditioned groups in comparison to HIR, which is favourable in achieving donor engraftment and suppressing a host response [24]. The downregulation seen at 2 weeks is in keeping with previous data from our lab [9], which could be because chemotherapy agents, which repress haematopoietic cell division may be suppressing inflammatory factor production, whilst after 2 weeks the incoming donor cells are able to repopulate and promote a pro-inflammatory environment. However, at 12 weeks once engraftment was complete, we noted sustained upregulation of *Il1b*, *Il6* and *Tnfa* in HIR mice, with a significant difference of *Il6* and *Tnfa* expression relative to NMC transplants, which did not initiate cytokine responses indicating a pro-inflammatory effect on the periphery with irradiation. HIR also upregulated the pro-inflammatory cytokine *Tnfa* in the spleen at 12 weeks post-transplant. The benefits of using a NMC busulfan transplant model pre-GBM implantation is that a slower chimerism model overtime promotes a more homeostatic environment for donor cells to engraft [19]. HIR models peak early with chimerism, but releases a cytokine cascade that could influence downstream immunobiology.

We subsequently analysed the mouse brain pre-GBM implantation for the same cytokines in order to determine neuroinflammation levels. When using HIR, mice are typically protected with a lead shield from the cranial apex to the cervical spine, ensuring no gamma rays target the brain. Our results showed that neither HIR nor NMC models demonstrated brain neuroinflammation. As expected, FC mice sustained elevated levels of *Ccl2*, *Il1b* and *Tnfa*. In FC transplant, this elevation of cytokines in the brain promotes monocyte-Mɸ infiltration and is the basis of haematopoietic stem cell gene therapy for neurological diseases [25].

In order to determine the baseline brain infiltration of peripheral lymphocytes in a homeostatic state, we used a untransplanted naïve control. In UnTx mice, we show the presence of a small proportion of leukocytes (5.6%) in the perfused brain made up almost exclusively of circulating lymphocytes, which probably fulfil an immune surveillance role [26]. In our HIR and NMC models post-BM transplantation, we also demonstrated similar percentage proportions of lymphocytes as a fraction of the total CD45^+^ population in the brain (4.3% and 3.2% respectively), but importantly no other immune cells were identified. Both models therefore appear to maintain a non-immunogenic state in the brain post-BM transplant. On the other hand, a fully myeloablative dose of busulfan (FC group) induces lymphocytosis in the brain, but also stimulates a significant surge of differentiated Mɸ into the brain with an overall larger population proportion of monocytes/Mɸ in comparison to microglia before recapitulation of GBM (Fig. 2). Our findings are in line with previous studies, achieving brain engraftment and differentiating Mɸ to display microglia characteristics [9, 18]. Elevated MCP-1 signalling [9] and a likely toxic effect of busulfan at these higher doses on brain microglia [18] lead to replacement of these cells with peripheral Mɸ. NMC busulfan on the other hand has been shown in a recent study to only have minor toxic effects or subsequent physiological effects on peripherally circulating and thymic memory T cells, indicating a more homeostatic model [27]. Using low-dose busulfan 20 mg/kg, conditioned hosts post-BM transplant demonstrates normal numbers of thymocytes at 6 weeks and then to a year post-BM reconstitution [27]. Furthermore, chimerised mice exhibit normal levels of proliferation using Ki67, peripheral CD4 naïve and CD4 memory T cells [27]. Further to this, we have also shown no change in T and B cell composition post-NMC busulfan BM transplantation to suggest recipient versus donor response. We cannot exclude that low-dose busulfan may target the turnover of brain microglia with Mɸ, but the reduced dose will limit the effects of busulfan beyond the BBB and our data suggest that this is minimal. Importantly, we do not see elevation of brain MCP-1 in the low-dose busulfan group (NMC), suggesting that this turnover is significantly reduced. No clear difference in brain immunophenotype was seen between NMC and HIR mice pre-GBM implantation.

When mice were implanted with GBM, we studied the contribution of lymphocytes in the brain in the different models at 14 weeks post-bone marrow transplantation, highlighting a slightly higher overall contribution of eosinophils in HIR mice. In addition to increased eosinophils, we also showed an elevated expression of *Il6* in the blood of HIR mice, which has previously been observed in human blood samples taken from asthmatic patients, with authors suggesting *Il6* to be constitutively synthesised and stored within eosinophils [28, 29]. Isolated studies have also reported eosinophilia secondary to irradiation [30, 31], which typically orchestrates a T cell response to cancer and indirectly stimulates *Il6* [32]. *Tnfa*, another cytokine of pro-inflammation, is typically reduced in the GBM microenvironment. However, our study and previous studies have shown that irradiating microglial cells increases expression of both *Il1b* and *Tnfa*, both of which contribute to a localised inflammatory response to foreign or tumour antigens [33–35]. Our findings demonstrate both an increased amount of differentiated Mɸ and *Tnfa* production in HIR-treated mice with tumours, thus potentially altering GBM outcomes in these mice. Importantly in NMC mice, there was no downstream impact of cytokine expression in the peripheral bloodstream on NMC mice. In this study, it is important to note the limitations of the GL-261 model when cultured in FCS, which could induce biased-FCS inflammatory reactions to GBM cells. Furthermore after 14 weeks or 2 weeks (day 14) post-GBM implantation, mice became very sick by day 15 and demonstrated an inconsistent analysis of brain immunophenotype due to the varying immune responses at a terminal stage of mouse survival.

Human studies using high and low doses of irradiation have previously demonstrated secondary inflammatory effects on T and B cells and dendritic cell (DC) function [36]. Essentially, radiation imbalances the immune system, and a spectrum of radiosensitivity exists ranging from the radiosensitive B cells through to the radioresistant memory T cells [37]. Initially, tissue damage occurs following irradiation resulting in a change in the redox, which primes the naïve recipient tissue microenvironment for immune activation; previous studies have shown this increases expression of MHC I and II, co-stimulatory molecules and chemokine receptors in preparation for an autoimmune response to foreign antigens [38–40]. As a result, a confounded environment would ensue. We show pro-inflammation in the peripheral blood of HIR mice, which expresses significant levels of *Il6* and *Tnfa* and increased levels of *Il1b*, and in the spleen significant upregulation of *Tnfa*. Taking this into account, the percentage proportions of CD45^+^ peripheral immune cells is lower in irradiated mice (50.6%) compared to both low-dose (NMC; 76.1%), consistent with a previous study [19] and high-dose (FC; 85.2%) busulfan-conditioned mice. We hypothesise that the peripheral immunogenic effects of HIR negatively impact upon immune cell trafficking into the brain, thus saturating the peripheral immune system with immune cells secondary to conditioning, allowing for less immune cells to target the implanted GBM.

## Conclusion

Adoptive transfer is a powerful tool to dissect the tumour-immune interactions. We have developed a novel non-myeloablative busulfan chimerism model and compared it to head-shielded irradiation. Both HIR and NMC achieve very similar levels of peripheral engraftment and both prevent BBB damage and inflammation in the brain. The main difference that exists is peripheral cytokine expression in the HIR model. Nonetheless, both models have drawbacks; non-myeloablative-conditioned mice take 12 weeks for maximal engraftment. HIR has shown increased pro-inflammatory cytokine expression post-conditioning and transplant that may lead to bias in immune cell infiltration and potentially perturbs any downstream inflammatory model. Overall, there is no clear answer either way which technique is better, as both possess drawbacks; nonetheless, the authors would recommend the NMC adoptive transfer model if the peripheral immune response remains an important question to be answered in the neurological study.

## Additional files

Additional file 1: **Figure S1.** Peripheral blood chimerism in all chimeric groups and unchimerised PEP-3 mice. a Increasing donor cell CD45.2 chimerism shown at 2, 4, 8 and 12 weeks after bone marrow (BM) transplant. b Peripheral blood chimerism in all four groups at different time points post-BM transplant (TIF 2493 kb)

Additional file 2: **Figure S2.** Organ chimerism in all four groups. Representative flow cytometry plots of bone marrow, spleen and brain (no tumour) of the same mouse in different bone marrow transplant groups and control. Brain samples of mice implanted with tumours (GBM) and relative donor infiltration/chimerism are shown on the bottom row (TIF 1910 kb)

Additional file 3: **Figure S3.** Gene expression of the brain at 12 weeks post-transplant without GBM. Brain samples without a tumour were analysed for the anti-inflammatory cytokines *Il10*, *Cxcl10*, *Il4* and *Ifna4*. All samples failed the Shapiro-Wilk normality test and were analysed using Kruskal-Wallis test with Dunn’s post hoc correction for multiple comparisons. No significant differences were noted between all samples. *Il4* demonstrated no expression in all samples and was excluded from the analysis (TIF 602 kb)

## Abbreviations

BBB: Blood-brain barrier
BM: Bone marrow
CNS: Central nervous system
DC: Dendritic cell
FC: Full conditioning
FCS: Foetal calf serum
GAMM: Glioma-associated macrophages and microglia
GAPDH: Glyceraldehyde 3-phosphate dehydrogenase
GBM: Glioblastoma
HIR: Head-shielded irradiation
HSCT: Haematopoietic stem cell transplantation
IFN-ɣ: Interferon gamma
IL-1β: Interleukin-1 beta
IL-6: Interleukin-6
IVC: Individually ventilated cages
MCP-1/CCL2: Monocyte chemoattractant protein-1
Mɸ: Macrophages
NMC: Non-myeloablative conditioning
PBS: Phosphate-buffered saline
RPMI: Roswell Park Memorial Institute
TNF-α: Tumour necrosis factor alpha
UnTx: Untransplanted
WBI: Whole-body irradiation
