# Supplementary figures and images for: Non-myeloablative busulfan chimeric mouse models are less pro-inflammatory than head-shielded irradiation for studying immune cell interactions in brain tumours
Source: J Neuroinflammation. 2019 Feb 5;16:25. doi: 10.1186/s12974-019-1410-y (PMC6362590; doi:10.1186/s12974-019-1410-y)

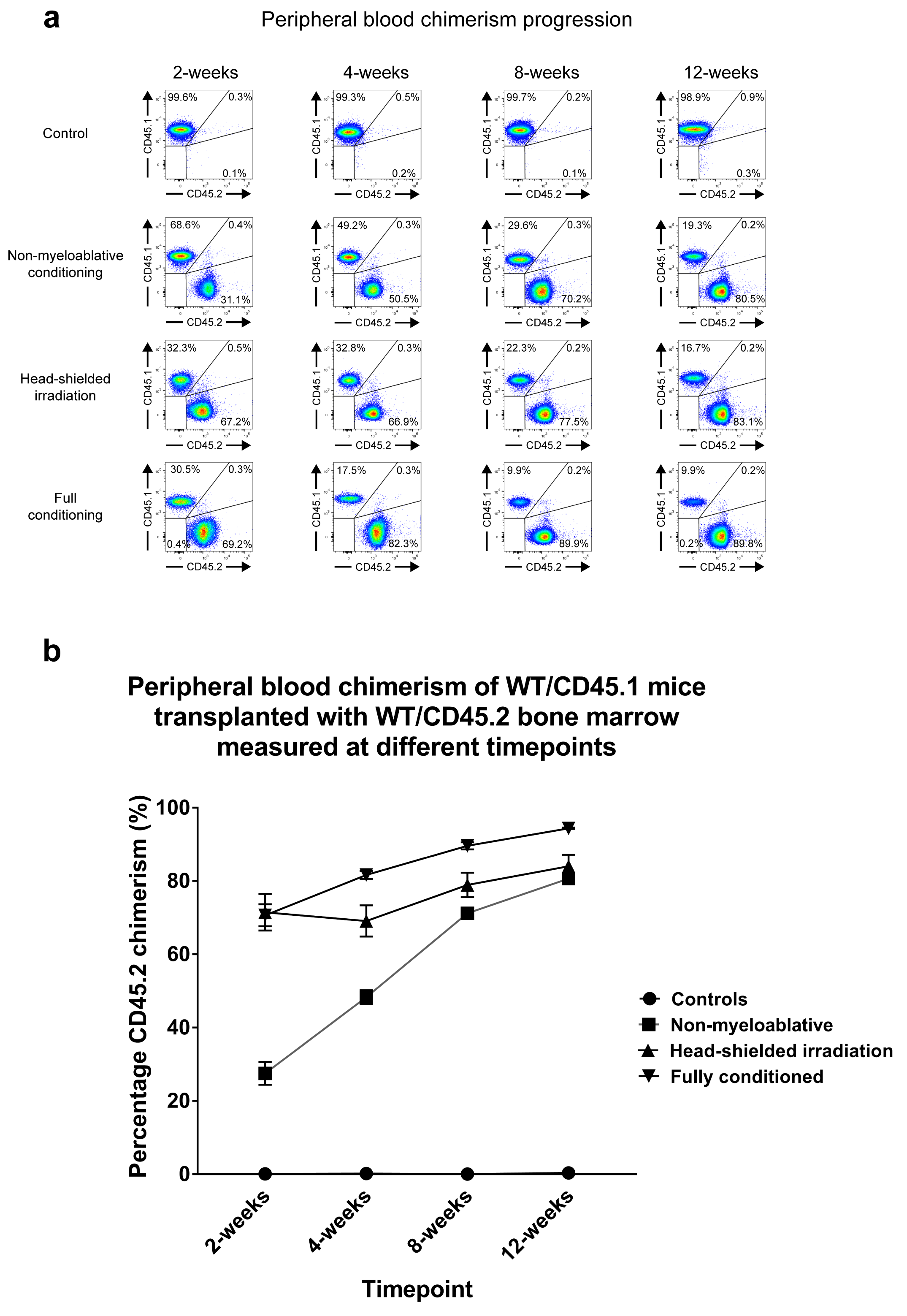

Supplement: Supplementary file 1 — Figure S1. Peripheral blood chimerism in all chimeric groups and unchimerised PEP-3 mice. a Increasing donor cell CD45.2 chimerism shown at 2, 4, 8 and 12 weeks after bone marrow (BM) transplant. b Peripheral blood chimerism in all four groups at different time points post-BM transplant (TIF 2493 kb) [file 12974_2019_1410_MOESM1_ESM.tif]

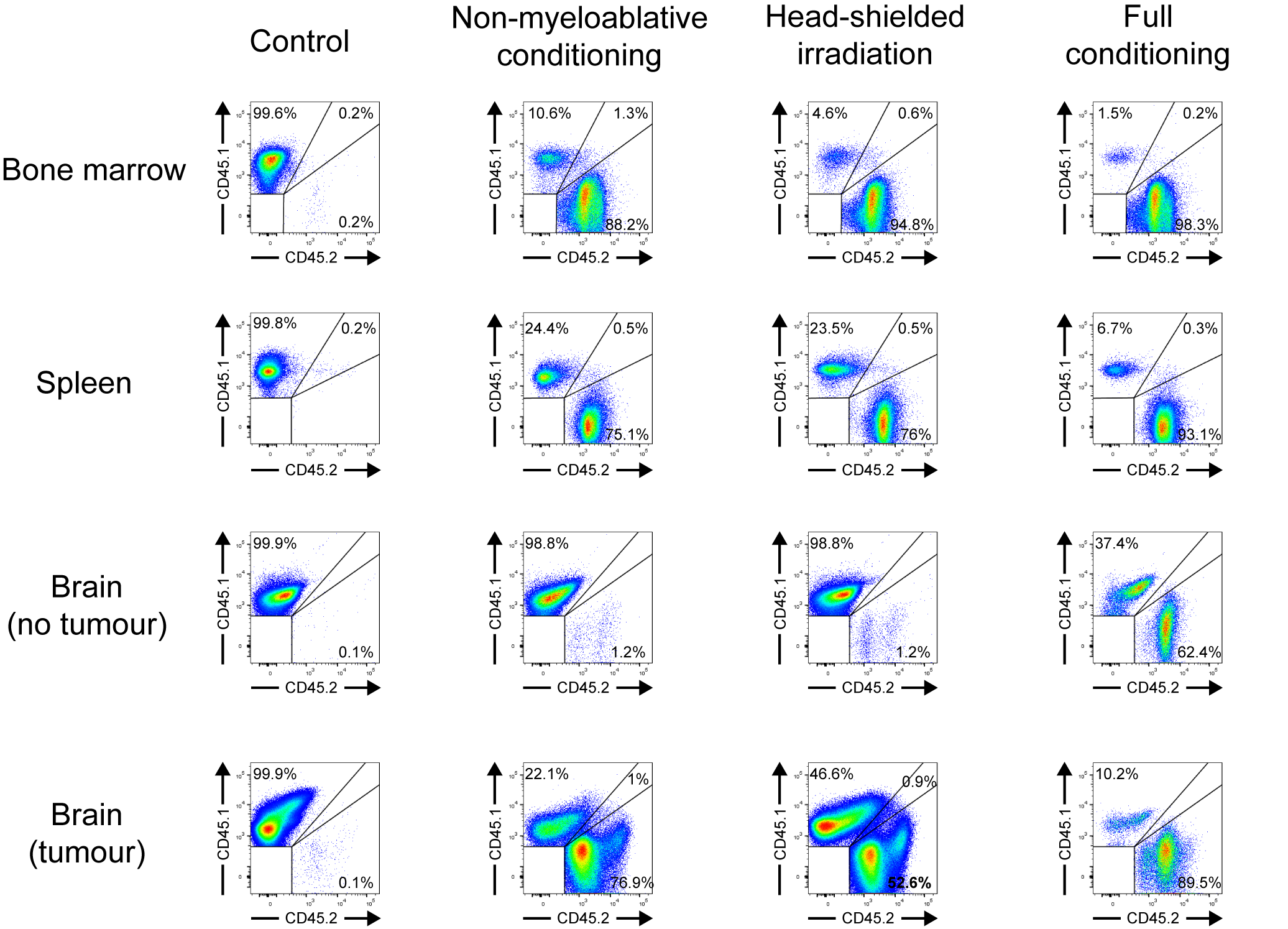

Supplement: Supplementary file 2 — Figure S2. Organ chimerism in all four groups. Representative flow cytometry plots of bone marrow, spleen and brain (no tumour) of the same mouse in different bone marrow transplant groups and control. Brain samples of mice implanted with tumours (GBM) and relative donor infiltration/chimerism are shown on the bottom row (TIF 1910 kb) [file 12974_2019_1410_MOESM2_ESM.tif]

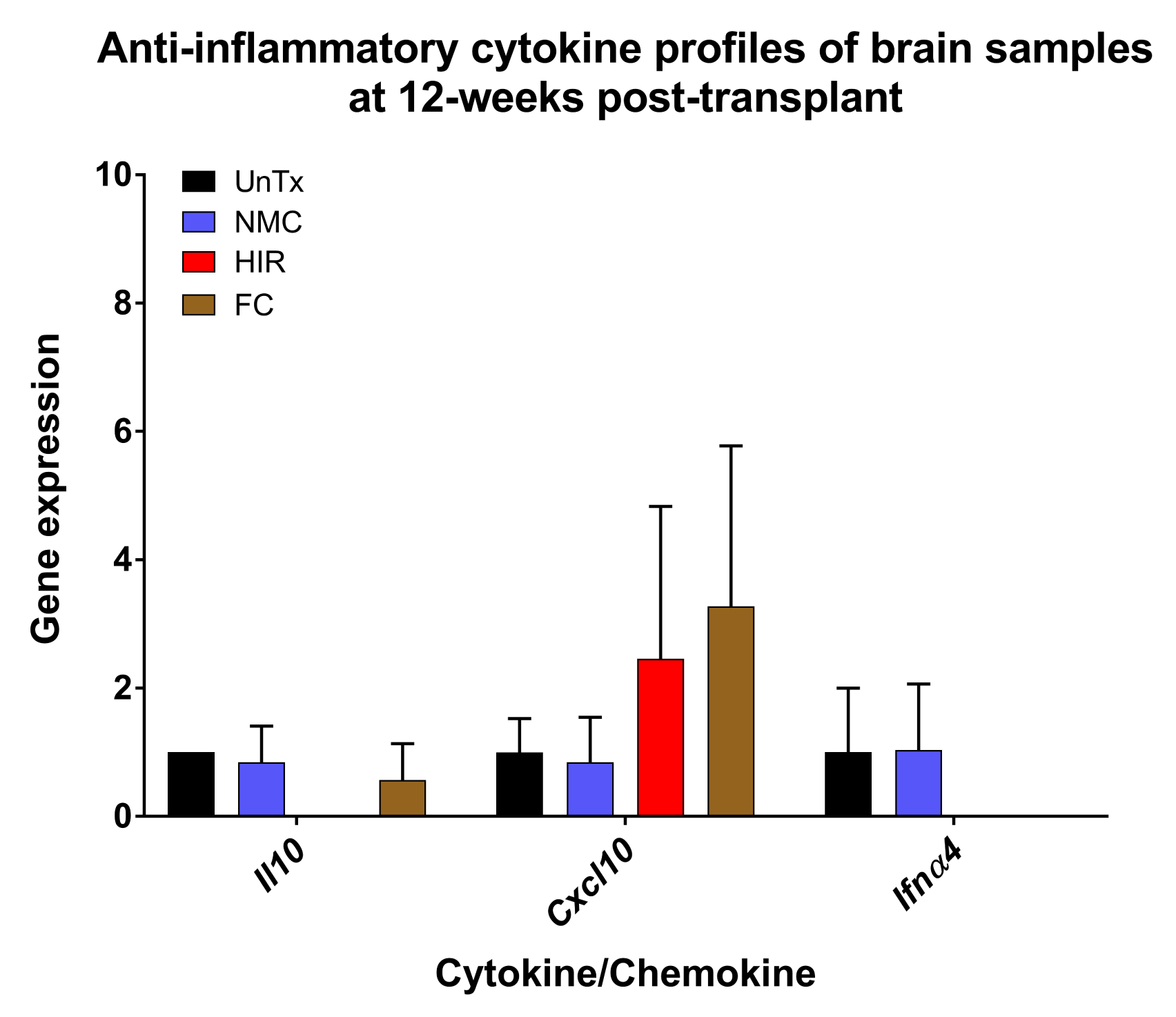

Supplement: Supplementary file 3 — Figure S3. Gene expression of the brain at 12 weeks post-transplant without GBM. Brain samples without a tumour were analysed for the anti-inflammatory cytokines Il10, Cxcl10, Il4 and Ifna4. All samples failed the Shapiro-Wilk normality test and were analysed using Kruskal-Wallis test with Dunn’s post hoc correction for multiple comparisons. No significant differences were noted between all samples. Il4 demonstrated no expression in all samples and was excluded from the analysis (TIF 602 kb) [file 12974_2019_1410_MOESM3_ESM.tif]
